# Macroscale Superlubricity Accomplished by Sb_2_O_3_-MSH/C Under High Temperature

**DOI:** 10.3389/fchem.2021.667878

**Published:** 2021-04-15

**Authors:** Kai Gao, Bin Wang, Asghar Shirani, Qiuying Chang, Diana Berman

**Affiliations:** ^1^State Key Laboratory of Tribology, Tsinghua University, Beijing, China; ^2^School of Mechanical, Electronic and Control Engineering, Beijing Jiaotong University, Beijing, China; ^3^Materials Science and Engineering Department, University of North Texas, Denton, TX, United States

**Keywords:** macroscale superlubricity, magnesium silicate hydroxide, Sb_2_O_3_, burnishing, high-temperature, tribology, tribochemistry

## Abstract

Here, we report the high-temperature superlubricity phenomenon accomplished in coatings produced by burnishing powders of antimony trioxide (Sb_2_O_3_) and magnesium silicate hydroxide coated with carbon (MSH/C) onto the nickel superalloy substrate. The tribological analysis performed in an open-air experimental setup revealed that with the increase of testing temperature, the coefficient of friction (COF) of the coating gradually decreases, finally reaching the superlubricity regime (the COF of 0.008) at 300°C. The analysis of worn surfaces using *in-situ* Raman spectroscopy suggested the synergistic effect of the inner Sb_2_O_3_ adhesion layer and the top MSH/C layer, which do not only isolate the substrate from the direct exposure to sliding but also protect it from oxidation. The cross-sectional transmission electron microscopy (TEM) and X-ray photoelectron spectroscopy (XPS) results indicated the tribochemically-activated formation of an amorphous carbon layer on the surface of the coating during sliding. Formation of the film enables the high-temperature macroscale superlubricity behavior of the material system.

## Introduction

Superlubricity [the coefficient of friction (COF) is <0.01], or the sliding regime with friction approaching almost zero values, is one of the research hotspots in the tribology field (Berman et al., 2018a). It was first proposed by Motohisa Hirano (Hirano and Shinjo, 1993) to describe a theoretical state: the friction force is extremely small or vanished between the tribopairs. So far, the superlubricity was mostly demonstrated for the laboratory-scale experiments, while use of the superlubricity in the large-scale industrial set-ups remains challenging. Previous studies demonstrated superlubricity mostly with 2D materials (Berman et al., 2018a), including graphite (Dienwiebel et al., 2004; Liu et al., 2012), graphene (Berman et al., 2015, 2019; Kawai et al., 2016), molybdenum disulfide (MoS_2_) (Martin et al., 1993; Li et al., 2017; Berman et al., 2018b), boron nitride (BN) (Zeng et al., 2013), black phosphorus (BP) (Wang et al., 2018a,b). The mechanism of the superblurcitiy that uses the easy shearing properties of 2D materials is also called structural superlubricity. In addition to two-dimensional materials, there are other ways to achieve superlubricity. For example, Li et al. (2014) tried to mix acids with glycerol or polyhydroxy alcohols that contained large amounts of hydroxyl groups indicating that the possible superlubricity mechanism was associated with the formation of a fluid-hydrated water layer between glycerol and water or polyhydroxy alcohol and water on the positively charged surfaces.

Meanwhile, most of the previous studies demonstrating superlubricity regime with solid lubricants focused on material systems that work at room temperature conditions. As a result, the justified cases of the superlubricity became very limited in terms of their applications. In efforts to solve the tribological challenges related to high temperature applications, focus was rather directed toward ternary oxides and carbides that are in general incapable of near-zero friction sliding (Gao et al., 2015; Aouadi et al., 2020). A possible solution is to enable adaptive behavior of materials under changes in the testing conditions which was successfully shown in case of graphite-MoS_2_ chameleon coatings (Shirani et al., 2020). The COF values were significantly reduced (down to 0.02 values) when testing the materials at elevated temperature conditions.

Here, we demonstrate that the superlubricity regime can be achieved at elevated temperature conditions in an open-air system by activating the formation of the low-friction tribofilm during sliding when using magnesium silicate hydroxide coated with a carbon layer (MSH/C). Magnesium silicate hydroxide is a novel lubricant additive and the main component of serpentine mineral with its tribological properties mainly being dependent on the release of Si-O, Mg-O, and -OH active groups that form a tribochemical layer during the friction process (Yu et al., 2010, 2013). To improve adhesion of the MSH/C film to the substrate material, we used antimony oxide (Sb_2_O_3_) powders (melting point: 656°C, boiling point: 1,425°C) (Jha et al., 2009). Though Sb_2_O_3_ is rarely used as an anti-wear or friction reduction additive, it is beneficial as a dopant to play a synergistic role with MoS_2_ in the solid lubricant coating (Zabinski et al., 1993, 1995; Hu et al., 2006). It was previously reported that the MoS_2_-Sb_2_O_3_-C film can change its surface with the environment changes to maintain good tribological performance by adapting to the testing conditions (Shirani et al., 2020). In another study it was found the COF of the sputter-deposited amorphous MoS_2_-Sb_2_O_3_-Au films decreases during sliding in a dry nitrogen environment as a result of MoS_2_ changing from amorphous to crystalline phase during this process (Scharf et al., 2010). Here, we also observe the synergistic effect of Sb_2_O_3_ and MSH/C as a result of tribochemically-activated material transformation into an amorphous carbon film which could support the easy sliding performance of the system. Our results propose a new solution to high temperature tribological issues.

## Experimental Procedure

### Materials and Film Preparation

Two types of the powders, MSH/C and antimony oxide (Sb_2_O_3_) were used to produce the coatings. The preparation details on the MSH/C powders could be found in our previous works (Gao et al., 2018a). Shortly, the powders were prepared by magnetic stirring a mixture of MgO and SiO_2_ as raw materials and oleic acid as a carbon source in a subcritical alkaline water environment at 300°C for 48 h. Antimony trioxide (Sb_2_O_3_) powders of ~5 μm in diameter were purchased from HiMedia (AR, CAS No. 1309-64-4).

The coatings used in this study were produced by burnishing powders onto a substrate surface. The burnishing process is a room-temperature mechanical, which uses balls or rollers to press lubricant powders on the surface (Hassan and Al-Bsharat, 1996). As for the film preparation, the specimen (Inconel IN718 substrate of 25 mm in diameter) was fixed on the rotating platform, and 0.011 g Sb_2_O_3_ was added during the rotation process. The substrate disk was polished before the burnishing process to the roughness Sa of 0.009 μm (Supplementary Figure 1). The rotation speed and force were adjusted during the burnishing process. Instead of using ball or pins, we used a lint free cloth to burnish. In the initial stage, the force and rotation speed were changed in the range of 1–5 N and 10–50 RPM, respectively. After obtaining the film, the force and rotation speed increased to 5–10 N, 100–200 RPM. As a result, Sb_2_O_3_ has been evenly distributed on the specimen surface thus forming a uniform adhesion layer. After obtaining the dense Sb_2_O_3_ layer on the surface, a layer of MSH/C (0.003 g) was uniformly spread on the surface by the same method.

### Friction Test

The friction tests were carried out using Nanovea T50 high temperature pin-on-disk tribometer. During the tests, the temperature of the samples and counterparts varied from 25°C (RT) up to 300°C using the high-temperature furnace operating under open air conditions. The friction pair was composed of a Si_3_N_4_ ball (diameter: 6 mm) and a sample of an MSH/C-based coating on the inconel (IN718) substrate (diameter: 25 mm). Before the test, the Si_3_N_4_ balls were cleaned by acetone followed by isopropyl alcohol. For the high-temperature test, the samples were heated at 2°C/min heating rate up to the target temperature and kept at the target temperature for at least 15 min prior to starting the tribo tests. Based on the preliminary tribological assessment of the burnished samples the testing parameters were adjusted as following: applied load was 0.59 N (corresponding to the maximum Hertz contact stress of 0.35 GPa), the rotational speed was 10 rpm, and the wear track radius was 4 mm. The used parameters are summarized in Table 1.

**Table 1:** The experimental details.

**Label**	**Load-Speed**	**Temperature**	**Revolutions**	**Materials**
Sb_2_O_3_	0.59 N-10 rpm	RT	500 cycles	Si_3_N_4_-IN718
Sb_2_O_3_	0.59 N-10 rpm	200°C	500 cycles	Si_3_N_4_-IN718
Sb_2_O_3_	0.59 N-10 rpm	300°C	500 cycles	Si_3_N_4_-IN718
Sb_2_O_3_-MSH/C	0.59 N-10 rpm	RT	500 cycles	Si_3_N_4_-IN718
Sb_2_O_3_-MSH/C	0.59 N-10 rpm	200°C	500 cycles	Si_3_N_4_-IN718
Sb_2_O_3_-MSH/C	0.59 N-10 rpm	300°C	500 cycles	Si_3_N_4_-IN718
Sb_2_O_3_-MSH/C	0.59 N-10 rpm	Variable Temp	500 cycles	Si_3_N_4_-IN718

### Characterization

After the test, the width of the wear scars and wear marks were directly observed and measured using a Zeiss optical microscope. The surface imaging of the samples before the measurements and after the tribotests was performed using Hitachi TM3030 scanning electron microscope (SEM) with energy dispersive x-ray spectroscopy (EDS) analysis. Raman characterization of the films was performed with Renishaw confocal Raman system (green laser) equipment with a home-built high temperature extension probe connected to the fiber optics attachment (the sampling beam size was 2 mm in diameter). Transmission electron microscopy (TEM) analysis was performed using FEI Technai G2 F20. X-ray photoelectron spectroscopy (XPS) analysis was performed using a PHI 5000 Versaprobe Scanning X-ray Spectrometer.

## Results and Discussion

**Figure 1:**
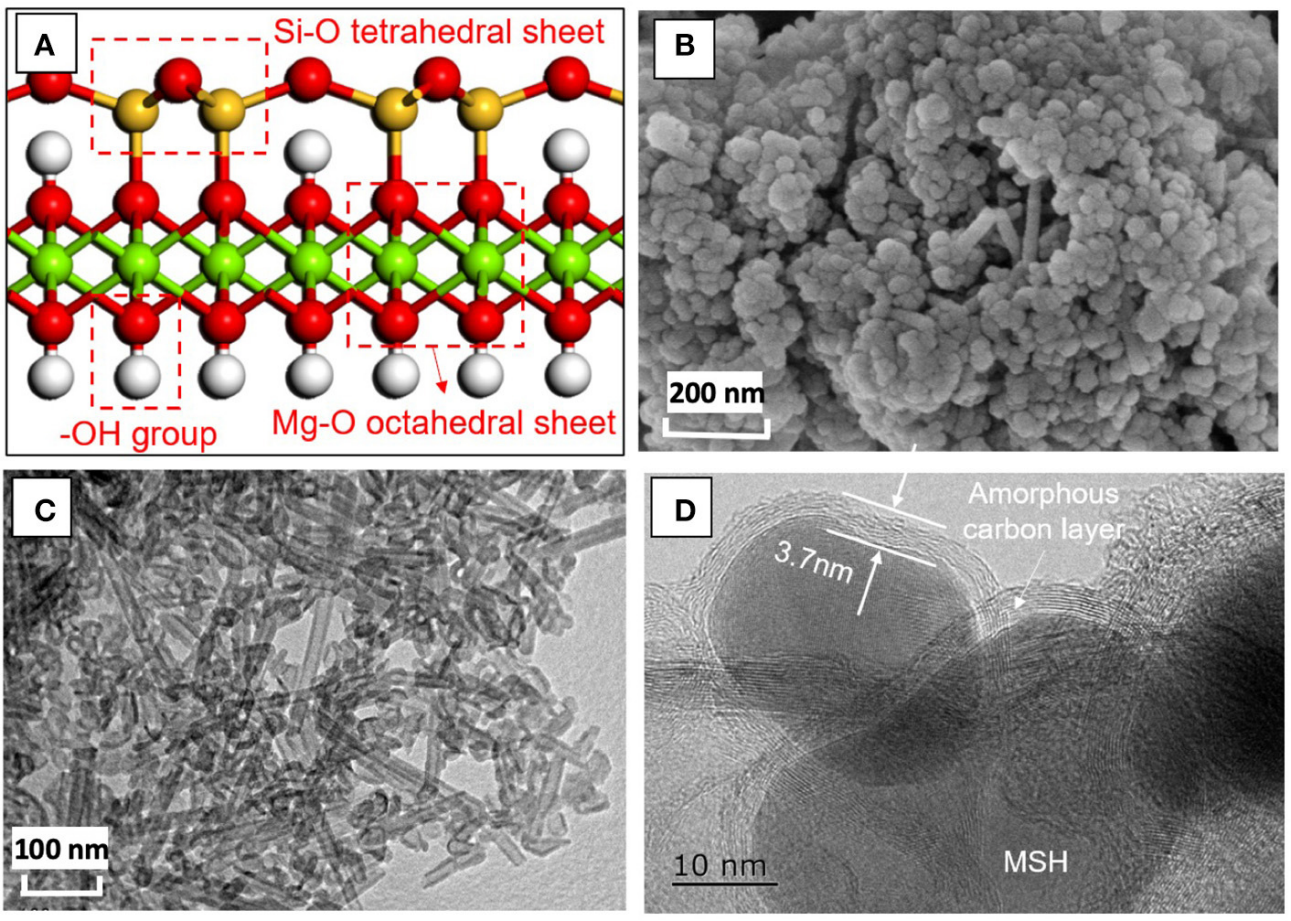
The **(A)** crystal structure, **(B)** SEM, **(C)** TEM images of MSH/C composite powders used in the solid film. **(D)** High resolution TEM analysis of the powders indicates presence of amorphous carbon layer on the MSH/C particle surface.

The coatings used in this study were designed using MSH/C powders (Figure 1). The final crystal structure (Figure 1A) of the powders indicates that they are composed of Si-O tetrahedral layer, Mg-O octahedral layer, and -OH group (Dixon, 1989; Zhao et al., 2012). Scanning electron microscopy (SEM) and TEM images indicate the the MSH/C poswders are the mixtures of granular and tubular shape particulates with diameter of ~30–50 nm and the tube length reaching hundreds of nanometers (Figures 1B,C). Transmission electron microscopy images also confirm that the particles are coated with an amorphous carbon layer with a thickness of several nanometers as indicated in Figure 1D.

**Figure 2:**
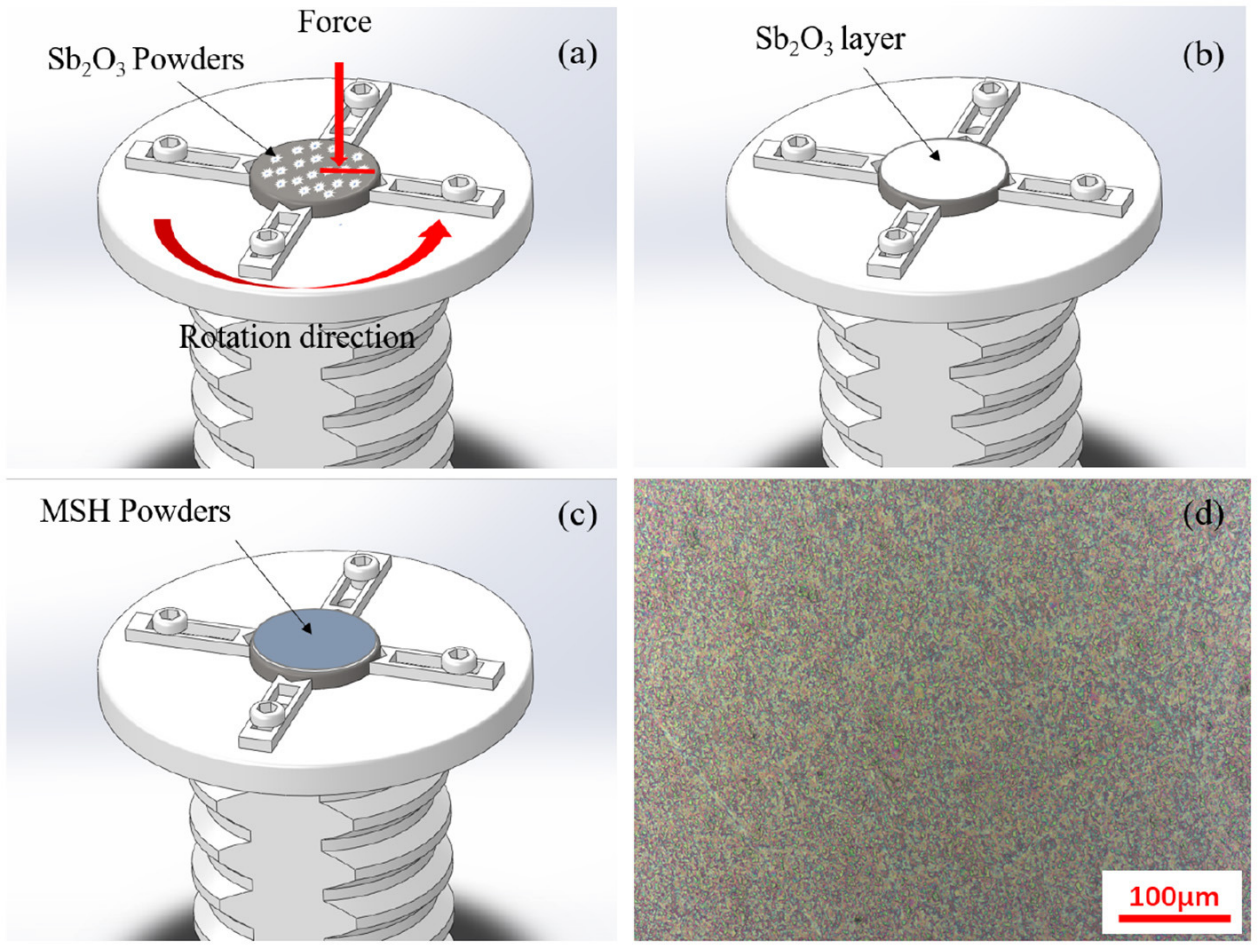
The diagram of the film preparation process: **(a)** antimony oxide powder is first used to create **(b)** a uniform adhesion layer that is then covered with **(c)** the MSH/C powder-based film. **(d)** SEM image indicates uniform coverage of the final sample surface.

The powders were used for coating the inconel substrates using the burnishing process. The schematic of the process and the final surface morphology are shown in Figures 2a–d. The average surface roughness of the coatings after burnishing was about 0.27 μm (Supplementary Figure 2). And the thickness of the prepared film was ~1 μm (Supplementary Figure 3). The hardness values for the inconel substrates and Sb_2_O_3_ and Sb_2_O_3_-MSH/C coatings were about 6.39, 2.517, and 2.196 GPa, respectively (tested by a nanoindentation tester, Supplementary Figure 4).

**Figure 3:**
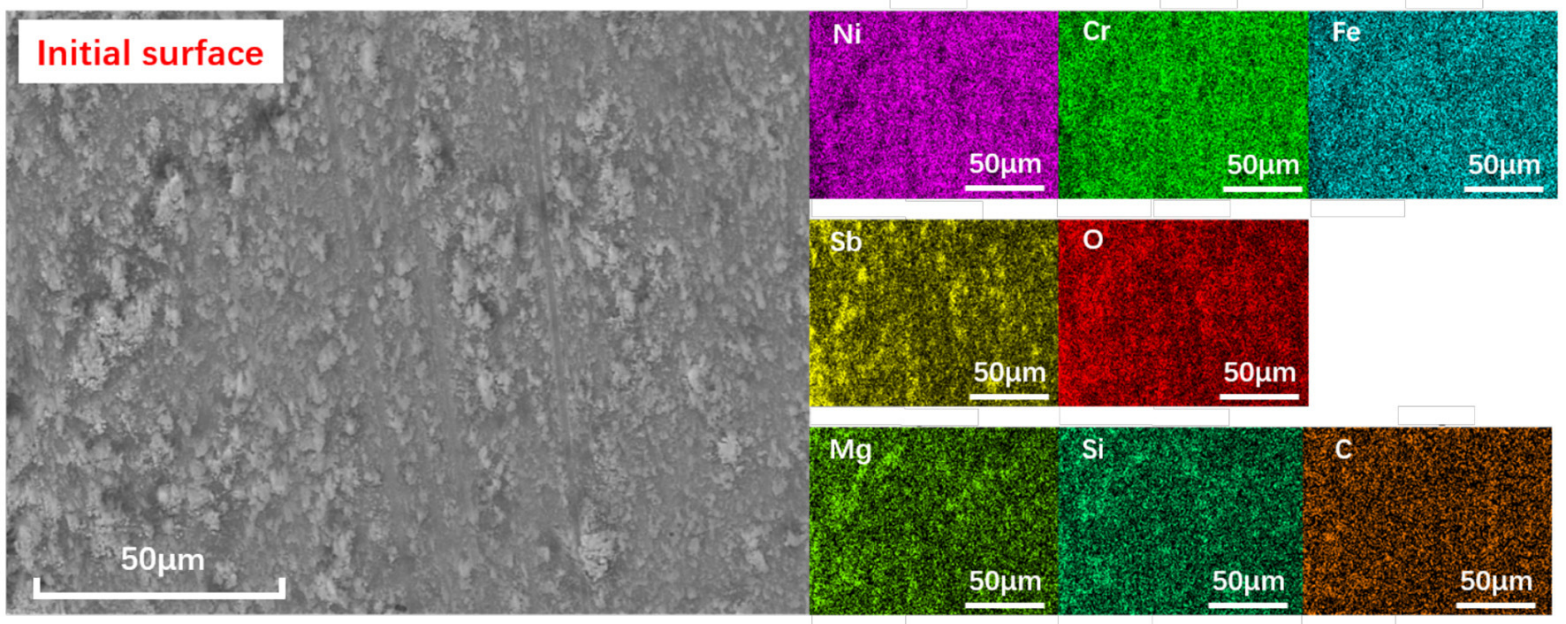
The SEM and the elemental mapping images of as-prepared Sb_2_O_3_-MSH/C surface. High uniformity of the particle distribution on the surface is observed.

The morphology and element distribution of as-prepared surfaces are shown in Figure 3. It can be seen that all the elements on the surface can be divided into three groups: Ni, Cr, and Fe belonging to the substrate material; Sb and O elements belonging to the Sb_2_O_3_ layer; and Mg, Si, O, and C belonging to the MSH/C composite nanoparticles. Note that Sb_2_O_3_ film prepared by the burnishing process is not uniformly distributed. Except for the layer on the surface, there are a large number of small Sb_2_O_3_ peaks in the form of aggregates, making the surface uneven. The presence of MSH/C composite nanoparticles is higher in the depression area composed of rough peaks, which is confirmed by the distribution of Mg and Si elements: except for the layer of MSH/C present on the surface, a significant increase in the content of Mg and Si elements was observed in the area surrounding the Sb_2_O_3_ peaks.

**Figure 4:**
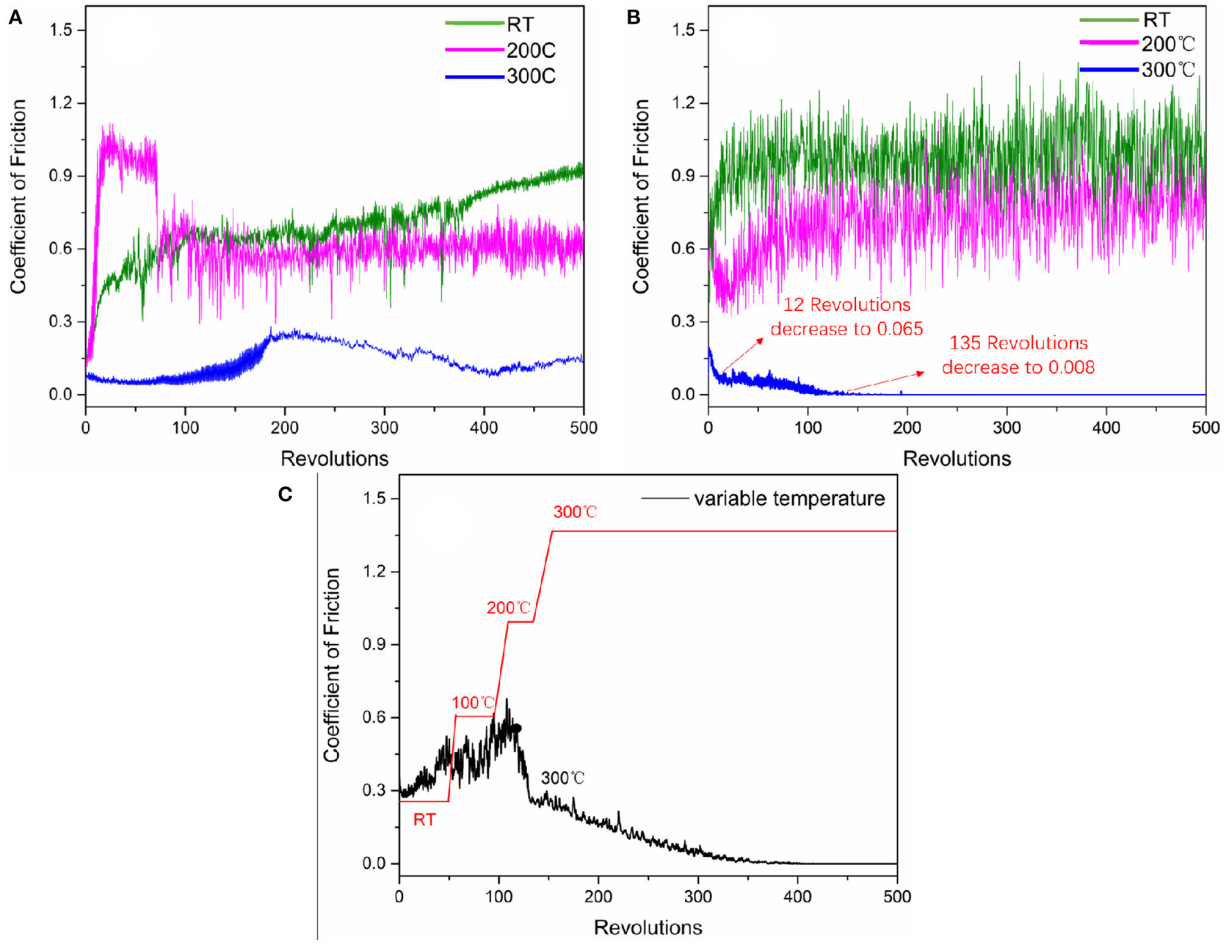
COF behavior of **(A)** Sb_2_O_3_, **(B)** Sb_2_O_3_-MSH/C coatings tested under different temperature comdition, and **(C)** Sb_2_O_3_-MSH/C coating continuously monitored during temperature ramping from the RT up to 300°C. Due to the limitation of furnace power, the heating rate for the temperature ranges of RT-100°C, 100–200°C, 200–300°C has been changed from 2.08°C/s to 1.11°C/s, and 0.83°C/s, respectively.

Designed coatings, after burnishing with Sb_2_O_3_ (labeled as Sb_2_O_3_) and after burnishing with Sb_2_O_3_ and MSH/C (labeled as Sb_2_O_3_-MSH/C) powders, were tested for their tribological performance. The COF results for the coatings tested at different temperatures are shown in Figures 4A,B. For the Sb_2_O_3_ coating, room temperature and 200°C tests resulted in considerably high COF values, 0.9 and 0.6 correspondingly. Meanwhile, further increase in the temperature to 300°C led to the COF reduction to 0.15. Such a decrease is attributed to elimination of water vapors adsorbed on the sample from the humid environment during burnishing process. After adding the MSH/C, the COF value was still high at room temperature and 200°C, reaching 0.95 and 0.8, respectively. However, as the temperature further increased to 300°C, the COF demonstrated rapid decrease after starting the test. After ~12 revolutions, the COF had dropped to 0.065. Further testing resulted in the COF showing a slow downward trend, and after 135 revolutions, the friction coefficient dropped to 0.008, thus entering the superlubricity region.

To further test the temperature sensitivity of the friction behavior of the coating, we continuously monitored the friction behavior while increasing the temperature from RT up to 300°C. The results indicated similar trend in the COF decrease under the elevated temperature conditions with friction approaching superlubricity values once the system reaches the higher temperature regime (Figure 4C). In the first 50 revolutions performed at RT, the COF continued to increase; ramping the temperature up to 100°C during additional 50 revolutions resulted in a more sporadic COF behavior around 0.4–0.5. Once the temperature reached 200°C, the COF dropped rapidly, changing from 0.63 to 0.25 within 38 revolutions. As the temperature increased further to 300°C over 150 revolutions, the COF continued to decrease steadily, and finally decreased to 0.008 at 400 revolutions, entering the superlubricity region.

**Figure 5:**
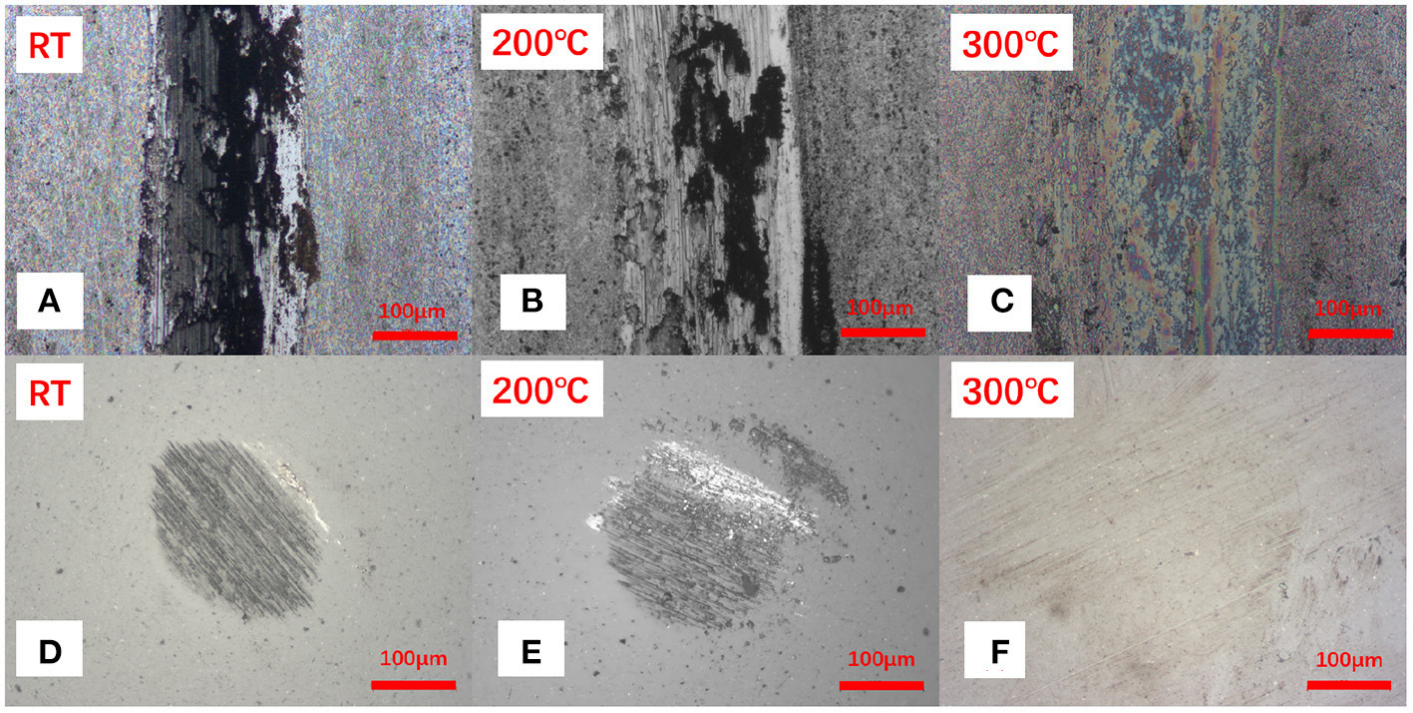
The optical images of **(A–C)** worn tracks, **(D–F)** corresponding ball worn surfaces for the Sb_2_O_3_-MSH/C sample after the tribotests performed under different temperature conditions.

**Figure 6:**
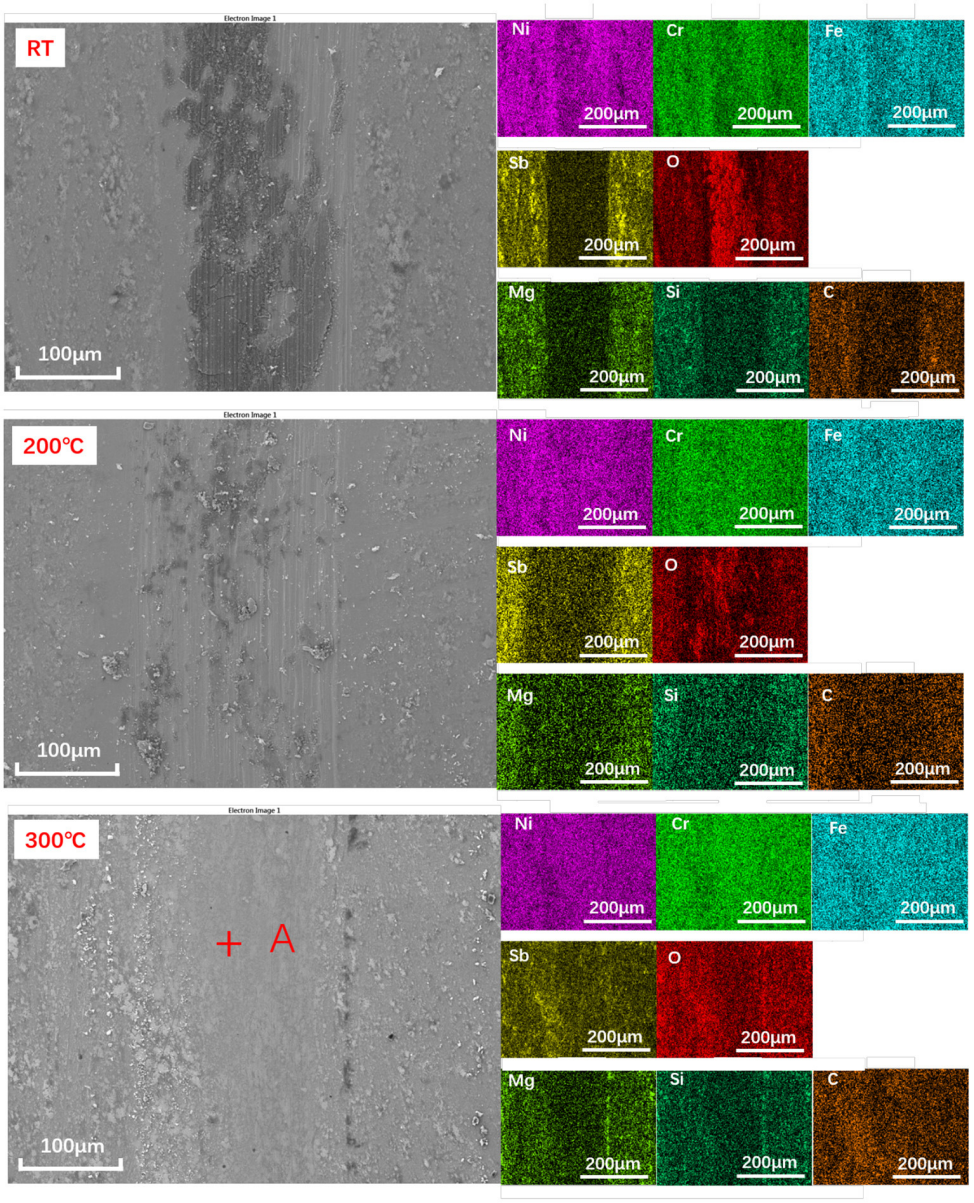
The SEM and EDS elemental mapping images of the Sb_2_O_3_-MSH/C wear track areas formed during the tribotests performed under different temperature conditions. Point A was selected for the XPS analysis.

To understand the origin of such behavior, we analyzed the wear tracks produced on the samples. Optical images of the wear scars and wear marks clearly show a difference when transitioning from the RT to the elevated temperature (Figure 5 and Supplementary Figure 5). While the high temperature tests indicate very mild wear of the materials, exposure of the samples to the lower temperature tests results in significant wear of the coatings. The micrographs suggest that under room temperature conditions the coating has been removed completely from the wear track during sliding resulting in the formation of a blackish film materials. The samples were further subjected to the SEM analysis suggesting that the blackish film is higher in the oxygen concentration, perhaps related to the metal oxide formed during sliding, rather than the coating materials (Figure 6 and Supplementary Figure 6).

**Figure 7:**
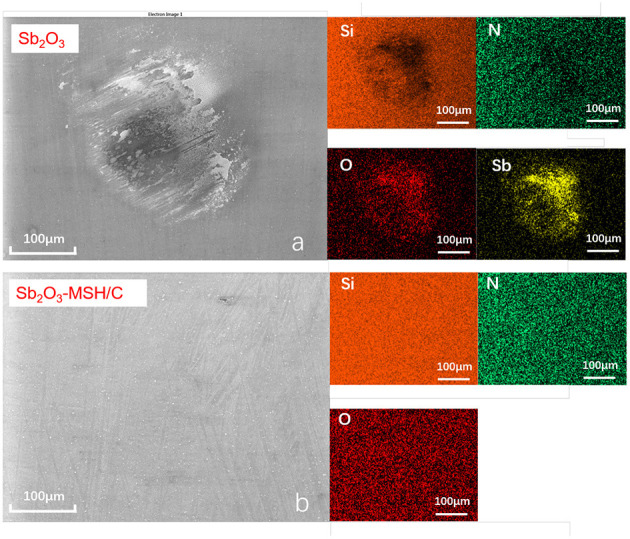
The SEM and the corresponding EDS elementap mapping images of the wearmarks for the silicon nitride balls after the tests performed at 300°C for **(a)** Sb_2_O_3_ and **(b)** Sb_2_O_3_-MSH/C samples.

As a result of lacking protective layer, large amounts of scratches appeared on the corresponding ball surface. As the temperature increased to 200°C, although more Sb, Mg, Si elements could be detected on the Sb_2_O_3_-MSH/C wear track area, the major part of the film has been worn out from the contact area, exposing the substrate surface and generating large amounts of oxidation products and scratches. However, when the tests were performed at 300°C, the wear track area was still covered by a Sb_2_O_3_-MSH/C layer even after the whole duration of the test, as shown in Figure 6. These results suggest that uniform coverage of the MSH/C film prevented exposure of the substrate to the sliding, thus inhibiting formation of the scratches and oxidation of the substrate materials. Evenmore, with the temperature increase, presence of the oxide products on the surface of the wear mark substantially decreased. This suggests that there is a synergy of protective properties of the Sb_2_O_3_ and MSH/C layers that do not only isolate the direct contact of friction pairs but also protect the substrate material from the oxidation. Analysis of the Si_3_N_4_ ball surfaces after the Sb_2_O_3_ tests indicated no obvious signs of wear but rather transfer of the protective coating to promote beneficial tribological characteristics of the films (Figure 7). Note that in case of Sb_2_O_3_-MSH/C, no obvious material transfer, such as Sb_2_O_3_ or carbon layer, is observed (Figure 7b and Supplementary Figure 7). This suggests that the composite film shows overall better integrity and the generated transfer film is weakly bonded to the silicon nitride surface and is easily wiped of during the post-processing. Therefore, the low-friction sliding at 300°C mostly occurs at the interfaces formed between MSH-generated carbon film and the silicon nitride ball surface. Similar wear-free behavior of the silicon nitride ball in contact with the diamond-like carbon (DLC) surface has been observed before by Jia et al. (1995).

Our results indicate that temperature plays an important role in the realization of superlubricity. Antimony trioxide (Sb_2_O_3_) itself still shows relatively high COF values suggesting that the Sb_2_O_3_ surface prepared by the burnishing process is rough with large number of Sb_2_O_3_ peaks being formed. Consequently, prolonged duration of the tests may lead to the complete removal of the top Sb_2_O_3_ layer and exposure of the metal substrate, which could cause high COF values and severe wear of the tribopairs. Observed decrease in the COF value at 300°C proved high temperature could be beneficial to the friction reduction.

**Figure 8:**
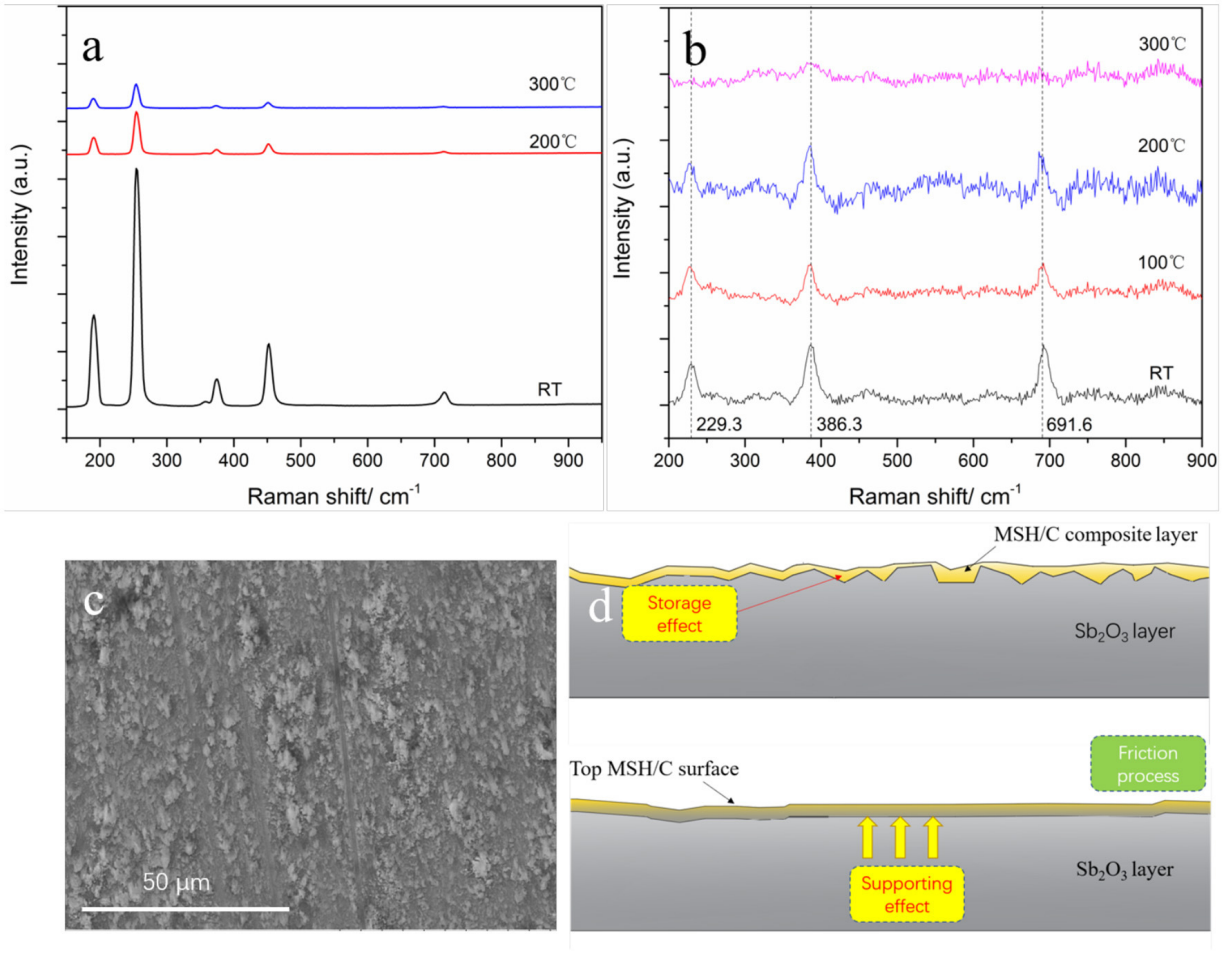
Raman spectrums of Sb_2_O_3_ **(a)** and MSH/C **(b)** under different temperatures (Excitation wavelength of 532nm) used for the tribological analysis, **(c)** the rough surface prepared by burnish process, and **(d)** the synergistic effect between the MSH/C layer and Sb_2_O_3_ layer.

We further probed the origin of temperature effect by *in-situ* Raman analysis of the coatings while heating (Figure 8). For Raman characterization of Sb_2_O_3_ film (Figure 8a), the measurement parameters and measurement positions were kept the same for the tested different temperatures. Resulting spectra indicate presence of six Raman peaks in the range of 150–950 cm^−1^: the peaks at 190 and 451 cm^−1^ come from the bending mode of Sb-O-Sb while the peaks at 255, 356, 373, and 714 cm^−1^ correspond to the stretching mode of Sb-O-Sb (Zeng et al., 2004). It can be seen that with the temperature increase, the intensity of each Raman peak gradually decreases, suggesting decrease in the corresponding vibrational groups. These results are in agreement with previously reported studies (Naidu et al., 2009) reporting that with the increase of temperature from room temperature and up to 400°C, the intensities of diffraction peak corresponding to Sb_2_O_3_ (110) crystal plane and (200) crystal plane gradually decrease, indicating that the crystal is gradually destroyed. The results suggest that such structural changes affect the adhesion of Sb_2_O_3_ to the metal substrate, thus reducing the direct contact of the tribopairs. Because of the Sb_2_O_3_ being used to improve the adhesion of the MSH/C layer to the substrate material, more MSH/C could be involved the friction reduction process. Thus, Sb_2_O_3_ provides support for the lubricant to remain at the sliding interface. Considering the combined storage and support role of Sb_2_O_3_ demonstrated by the presence of the rough peaks on the MSH/C powder shown in Figures 8c,d, the worn surface could still be covered by a layer of lubricant. Meanwhile, the addition of top MSH/C also protects the bottom Sb_2_O_3_ layer, which, as reflected by the mapping results at 200°C, results in more Sb being detected on the Sb_2_O_3_-MSH/C than on the Sb_2_O_3_ surface thus supporting the findings in Figure 6 and Supplementary Figure 6.

We evaluated the necessity of the MSH/C presence for achieving superlubricity under high temperature conditions. The Raman results of MSH/C under different temperatures reflected the changes in the MSH/C crystal structure upon temperature increase, as shown in Figure 8b. The -O-H vibration peak located at 229 cm^−1^ and the Si-O-Si peak located at 691 cm^−1^ disappeared after temperature reaching 300°C. Besides, the intensity of the SiO_4_ symmetrical stretching vibration peak at 386 cm^−1^ also decreased significantly. This indicates that the -O-H and Si-O bonds in the MSH/C powders are destroyed with the temperature increase, thus leading to the release of a large amount of Si-O and -OH groups. The TG-DSC results in our pervious work also confirmed the release of -OH group (also called as the stuctural water) with the increase of temperature (Gao et al., 2018b). These active groups released from the MSH/C powders are the key reason for their excellent tribological performance (Chang et al., 2017, 2019). Local heating induced by friction is expected to further promote the decomposition of the MSH/C and the release of various active groups. Considering the mild tribological test condition (0.59 N−10 rpm) used in our study, the MSH/C could present the best performance under 300°C since the heat conduction energy will make up for the lack of friction energy.

**Figure 9:**
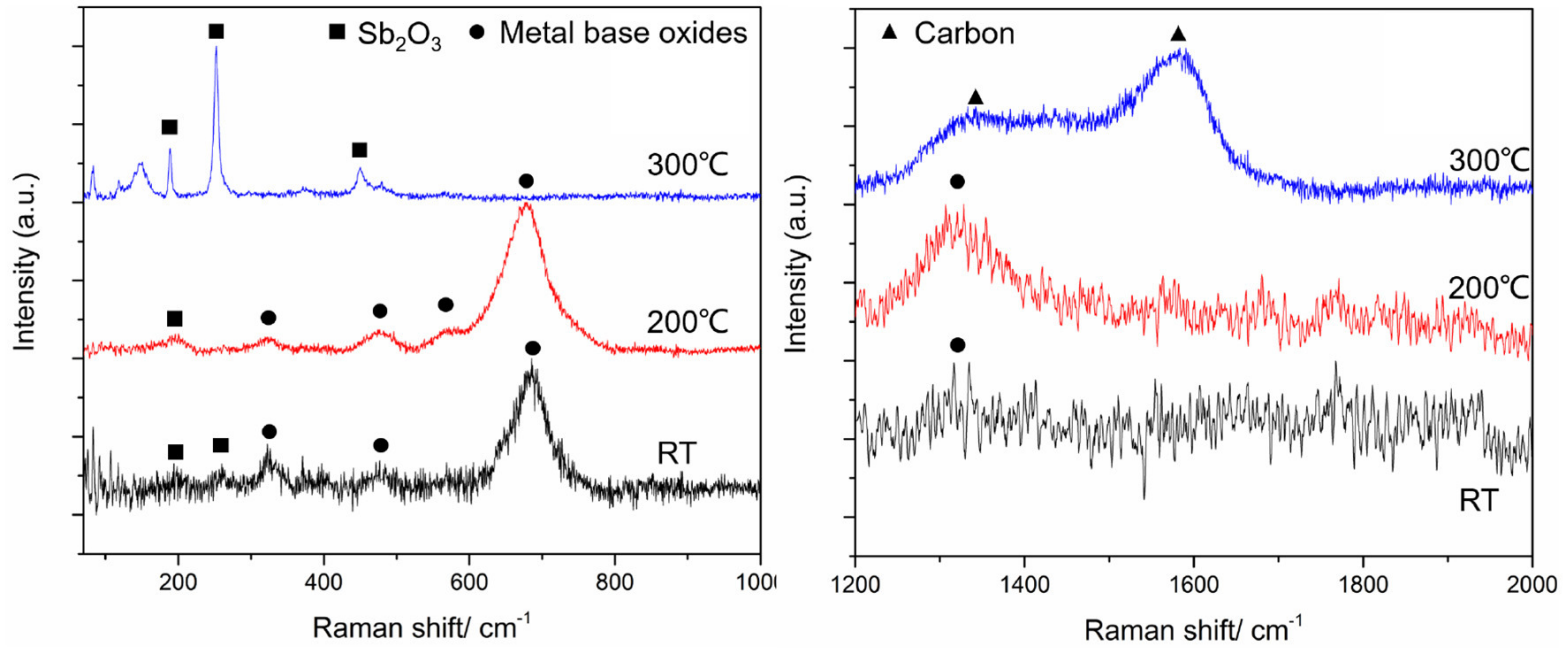
Raman results of the worn Sb_2_O_3_-MSH/C surfaces under different temperature conditions.

In addition to the analysis of the properties of the powders at different temperatures, we have carried out a more in-depth analysis of the coating surfaces after the tests. The Raman results of worn surfaces under different temperatures are shown in Figure 9. It can be noticed that under RT and 200°C, due to the large removal of the Sb_2_O_3_-MSH/C layer in the friction process, the metal substrate is exposed to sliding causing formation of oxides as it is confirmed by the presence of various metal oxide Raman peaks, including iron oxide (323 cm^−1^) (de Faria et al., 1997), nickel oxides (476, 560 cm^−1^) (Nan et al., 2006; Kumar et al., 2020), and chromium oxides (680 cm^−1^) (Guinneton et al., 2005). Besides, the Sb_2_O_3_ peak located at 190 cm^−1^, a new peak at 255 cm^−1^ also appeared, though its intensity is weak. No carbon or MSH related Raman peaks could be found on the worn surfaces. However, in case of 300°C test, strong Sb-O vibration peaks located at 190 and 255 cm^−1^ appeared, which further proved better integrity of the coating at higher temperature and better survivability of Sb_2_O_3_ on the metal surface. Meanwhile, during sliding at the lower temperatures, Sb_2_O_3_ does not have strong enough adhesion to the substrate and the coating does not remain intact during sliding. Importantly, high temperature test at 300°C also indicated presence of the carbon G peak and D peak located at 1,583 and 1,340 cm^−1^ respectively, that were not observed for the coatings heated but without being subjected to the shear stresses. It is worth to mention that the D peak shifts toward the low wavenumber with the influence of the Si element (Veres et al., 2006).

**Figure 10:**
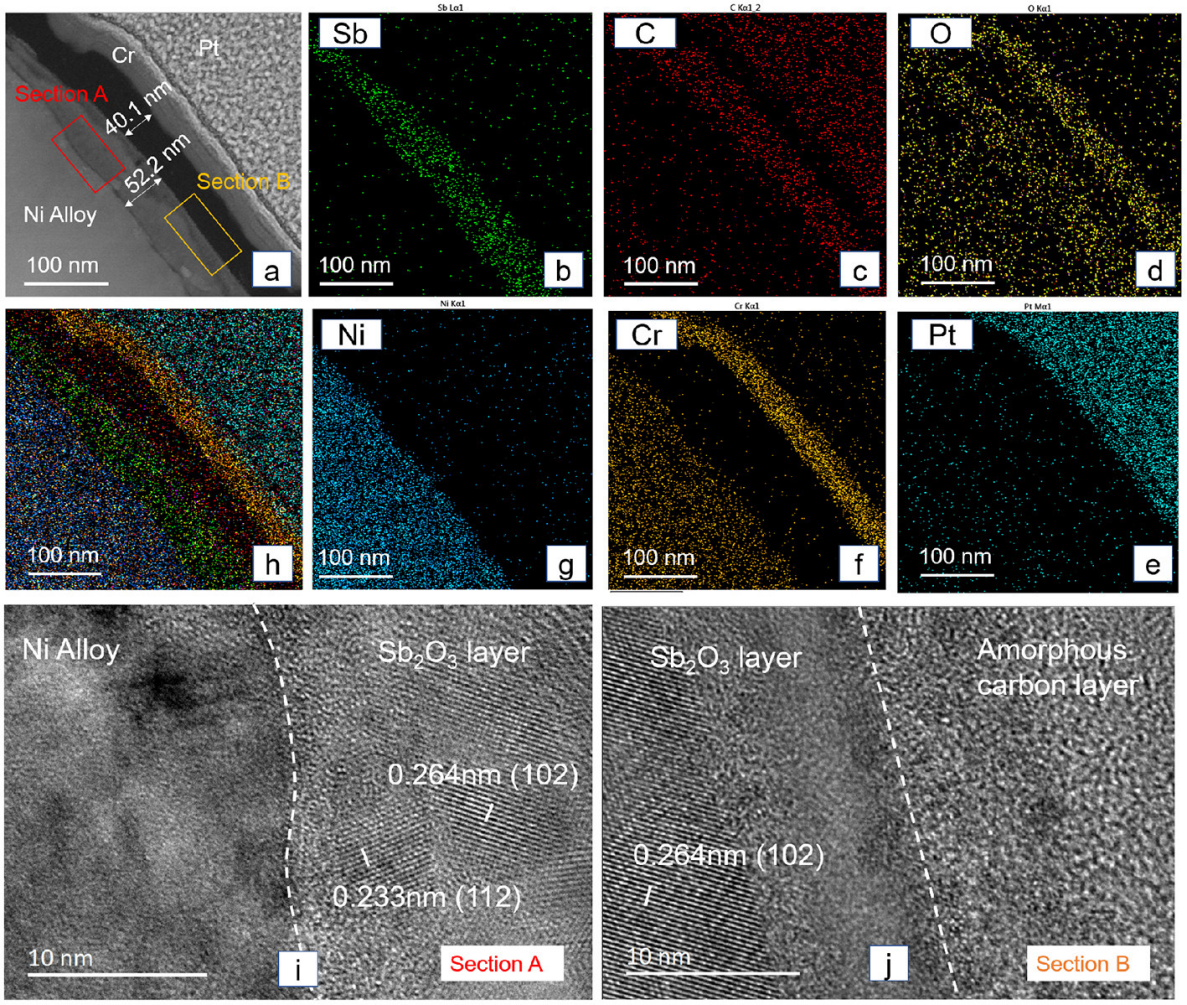
Cross-sectional TEM image of the Sb_2_O_3_-MSH/C surface after the test under 300°C **(a)**, the EDS elemental mapping of the cross-sectional area **(b–h)**, and High-resolution TEM image is taken from the interface area of different layers **(i, j)**, which is also marked as section A and section B in **(a)**. The Pt and Cr protective layers were deposited to protect the tribofilm from the damage during TEM specimen preparation.

The structure and composition of the tribofilm formed on the Sb_2_O_3_-MSH/C coating at 300°C were analyzed using the TEM analysis. For this, we use the Focused Ion Beam (FIB) technology to prepare a samples from the worn surface of Sb_2_O_3_-MSH/C after the test (Figure 10). The cross-sectional TEM image in Figure 10a and mapping results in Figures 10b–h indicate the existence of a Sb_2_O_3_ layer and an amorphous carbon layer with thicknesses reaching 52.2 and 40.1 nm, respectively. In contrast to the layered structure of the carbon layer observed in Figure 1D, the formed tribofilm is mostly of the amorphous nature. The High-resolution TEM image of Section A in Figure 10i shows the borderline between alloy base and Sb_2_O_3_ layer. Nanocrystalline structure and amorphous structure both existed in this Sb_2_O_3_ layer. Interestingly, the border section is occupied by the amorphous Sb_2_O_3_, not only in Section A but also in Section B shown in Figure 10j, which is the transition region between the Sb_2_O_3_ layer and amorphous carbon layer.

**Figure 11:**
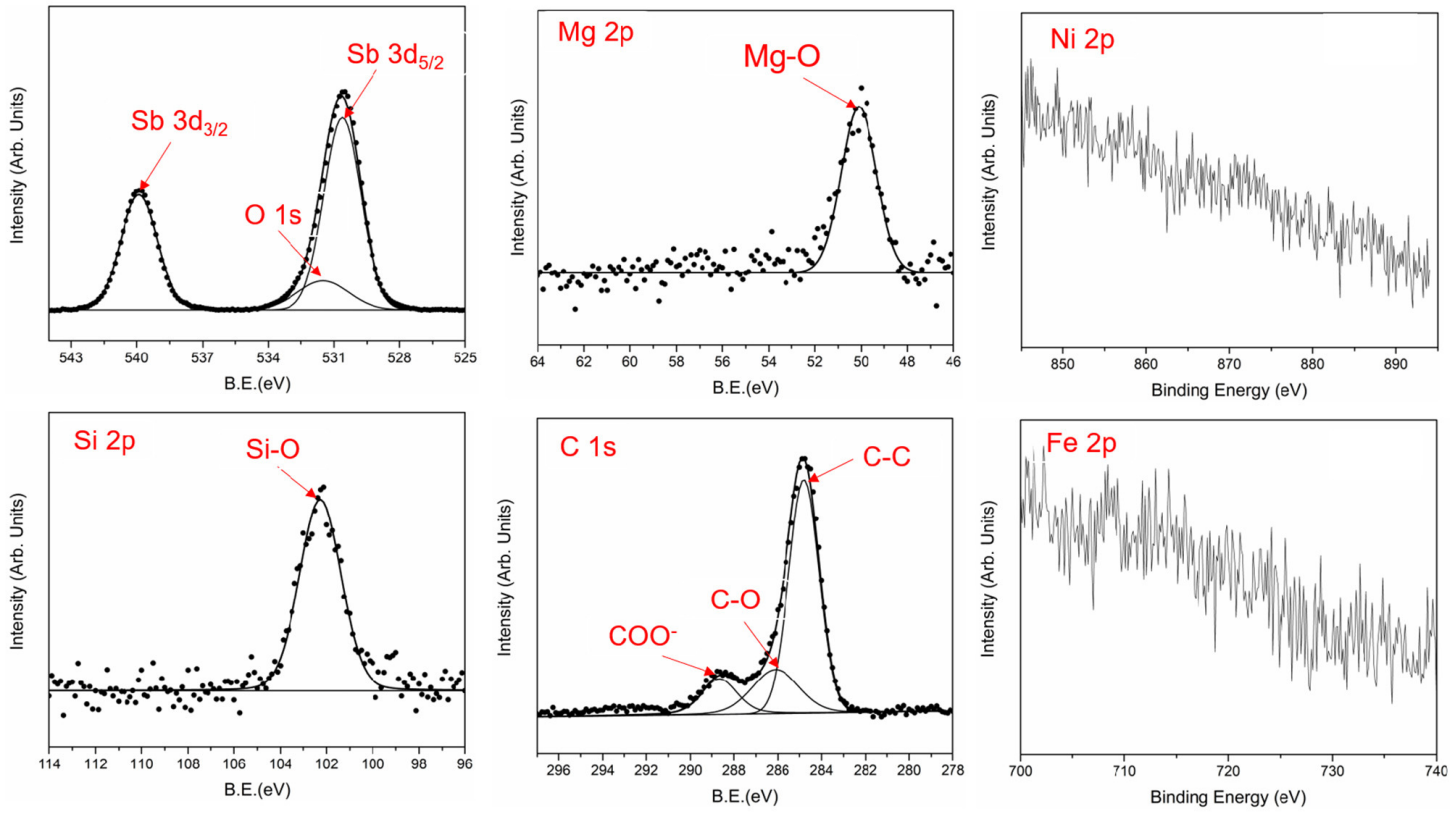
XPS spectra of the elements on the wear track area of Sb_2_O_3_-MSH/C under 300°C (point A in Figure 6).

The TEM results of the wear track were complemented by the XPS characterization of the tribofilm surface (Figure 11). There was no obvious signal of Fe, Ni and other elements belonging to the metal substrate in Figure 11, which indicates the metal matrix is not exposed during the friction process. The worn surface is mainly composed of C, O, and Sb elements with the elemental concentration (at.%) of 24.7, 62.2, and 7.6%, which is in agreement with the TEM results: an outer carbon layer, an inner Sb_2_O_3_ layer. The amorphous carbon layer in Figure 10j is made up of C-C, C-O, and COO^−^ as the C 1s peak can be fitted by three peaks at 284.8, 286.1, and 288.6 eV (Grzybek et al., 2004; Morent et al., 2008; Cai and Zhang, 2013; Samanta et al., 2019). The C-C bond only accounted for 66.1%, which indicates the oxidation reaction takes place in the process of high temperature friction resulting in about third of C atoms being bonded with O atoms to form C-O and O-C=O groups. The O 1s peak at 531.5 eV is covered by Sb 3d_5/2_ at 530.6 eV, but it can be separated by the rules, such as the equal half peak width, the peak distance (9.3 eV), and the relative area ratio (1.5) between Sb 3d_5/2_ peak and Sb3d_3/2_ peak that is centered at 539.9 eV (Honma et al., 2000; Huang and Ruiz, 2005). In addition, Mg and Si are also detected on the surface of the tribofilm, though at much lower concentration: Mg 2p 2.1%, Si 2p 3.2%, existing in the form of Si-O-C and Mg-O (Ardizzone et al., 1997; Jantschner et al., 2015; Zhang et al., 2020), which indicates the decomposition of in the friction process.

Overall, the characterization results suggest that during sliding the carbon initially covering the MSH surface has been tribochemically converted to a more disordered amorphous carbon layer protecting the underlying substrate. At the same time, the structural transformation has been accompanied by the oxidation induced by the open air conditions and MSH chemistry and accelerated by high temperature. Previously it has been shown that during sliding the MSH crystals tend to decompose, releasing Si-O, Mg-O, and -OH active groups which could promote formation of the oxide layer as the lubricating oil addtive (Wang et al., 2020). Here, in dry sliding conditions, these MSH-based active groups could cause the formation of the Si-O-C bonds (Jantschner et al., 2015). The tribochemically-activated formation of the composite amorphous carbon layer doped with Si and O on the surface of Sb_2_O_3_ is the key to reducing of the COF and achieving the superlubricity at 300°C, which has been confirmed earlier (Jantschner et al., 2014, 2015; Evaristo et al., 2016). The thermomechanical process also promotes healing of the creacks and defects in the film and improves the coating resistance to fracture and fatigue in the high-temperature contact. M. Evaristo et al. found that the incorporation of Si and O in the amorphous carbon layer can reduce the friction coefficient from 0.17 to 0.034 (Evaristo et al., 2016). O. Jantschner et al. pointed out that the addition of a small amount of Si (<5 at.%) to the amorphous carbon coating at 240–450°C also beneficially affects the tribological performance of the film [the COF was reduced from 0.3 (amorphous carbon) to <0.02] (Jantschner et al., 2014, 2015). Although the mechanism of friction reduction with Si and O doping is not clear, it is assumed the formation of Si-O-C compounds in the sliding surface could help protect the surface near C=O, thus inducing easier sliding at the interfaces (Jantschner et al., 2015).

## Conclusion

In conclusion, we designed a new MSH/C-based coating using burnishing process. While the friction remained relatively high during the room temperature tests, our analysis indicated excellent tribological performance of the coating when tested at elevated temperature of 300°C. The wear track analysis suggests that the origin of such high temperature behavior of the coating can be attributed to the formation of low-friction tribofilm that is enabled by tribochemically-induced formation of carbon layer on the surface of the coating. This film provides better protection of the surface during sliding and prevents material wear or transfer to the counterpart surface.

High temperature *in-situ* Raman analysis of the films further supported the findings of the study suggesting that the superlibric nature of the coating is a synergistic effect of the antimony oxide adhesion layer, tribochemically-active MSH powders, and tribochemically-generated surface carbon film.

Our results provide new insights into high temperature lubrication systems that can be easily self-repaired by supplying the materials to the sliding contacts through the burnishing process.

## Data Availability Statement

The raw data supporting the conclusions of this article will be made available by the authors, without undue reservation.

## Author Contributions

KG: investigation, data curation, and writing. BW and AS: data curation. QC: resources. DB: supervision, investigation, conceptualization, and writing. All authors contributed to the article and approved the submitted version.

## Conflict of Interest

The authors declare that the research was conducted in the absence of any commercial or financial relationships that could be construed as a potential conflict of interest.

## References

[B1] Aouadi S. M. Gu J. Berman D. (2020). Self-healing ceramic coatings that operate in extreme environments: a review. J. Vac. Sci. Technol. A 38:050802. 10.1116/6.0000350

[B2] Ardizzone S. Bianchi C. L. Fadoni M. Vercelli B. (1997). Magnesium salts and oxide: an XPS overview. Appl. Surf. Sci. 119, 253–259. 10.1016/S0169-4332(97)00180-3

[B3] Berman D. Deshmukh S. A. Sankaranarayanan S. K. R. S Erdemir A. Sumant A. V. (2015). Macroscale superlubricity enabled by graphene nanoscroll formation. Science 348, 1118–1122. 10.1126/science.1262024 25977372

[B4] Berman D. Erdemir A. Sumant AV. (2018a). Approaches for achieving superlubricity in two-dimensional materials. ACS Nano. 12, 2122–2137. 10.1021/acsnano.7b09046 29522673

[B5] Berman D. Mutyala K. C. Srinivasan S. Sankaranarayanan S. K. R. S. Erdemir A. Shevchenko E. V.. (2019). Iron-nanoparticle driven tribochemistry leading to superlubric sliding interfaces. Adv. Mater. Interfaces 6:1901416. 10.1002/admi.201901416

[B6] Berman D. Narayanan B. Cherukara M. J. Sankaranarayanan S. K. R. S. Erdemir A. Zinovev A.. (2018b). Operando tribochemical formation of onion-like-carbon leads to macroscale superlubricity. Nat. Commun. 9:1164. 10.1038/s41467-018-03549-6 29563513 PMC5862981

[B7] Cai C. C. Zhang M. X. (2013). XPS analysis of carbon and oxygen in coking coal with different density intervals. Appl. Mech. Mater. 347–350, 1239–1243. 10.4028/www.scientific.net/AMM.347-350.1239

[B8] Chang Q. Rudenko P. Miller D. J. Wen J. Berman D. Zhang Y.. (2017). Operando formation of an ultra-low friction boundary film from synthetic magnesium silicon hydroxide additive. Tribol. Int. 110, 35–40. 10.1016/j.triboint.2017.02.003

[B9] Chang Q. Y. Wang B. Gao K. (2019). Pressure-dependent anti-wear mechanisms of synthetic magnesium silicate hydroxide nanoparticles. Tribol. Int. 135, 230–236. 10.1016/J.TRIBOINT.2019.03.016

[B10] de Faria D. L. A. Venâncio Silva S. de Oliveira M. T. (1997). Raman microspectroscopy of some iron oxides and oxyhydroxides. J Raman Spectrosc 28, 873–878. 10.1002/(SICI)1097-4555(199711)28:11<873::AID-JRS177>3.0.CO;2-B

[B11] Dienwiebel M. Verhoeven G. S. Pradeep N. Frenken J. W. M. Heimberg J. A. Zandbergen H. W. (2004). Superlubricity of graphite. Phys. Rev. Lett. 92:126101. 10.1103/PhysRevLett.92.126101 15089689

[B12] Dixon J. B. (1989). Kaolin and serpentine group minerals. Miner. Soil Environ. 1, 467–519. 10.2136/sssabookser1.2ed.c10

[B13] Evaristo M. Azevedo R. Palacio C. Cavaleiro A. (2016). Influence of the silicon and oxygen content on the properties of non-hydrogenated amorphous carbon coatings. Diam. Relat. Mater. 70, 201–210. 10.1016/j.diamond.2016.10.024

[B14] Gao H. Otero-de-la-Roza A. Gu J. Stone D. Aouadi S. M. Johnson E. R.. (2015). (Ag,Cu)-Ta-O ternaries as high-temperature solid-lubricant coatings. ACS Appl. Mater. Interfaces 7, 15422–15429. 10.1021/acsami.5b03543 26106877

[B15] Gao K. Chang Q. Wang B. Zhou N. Qing T. (2018a). Synthetic magnesium silicate hydroxide nanoparticles coated with carbonaceous shell in subcritical water condition. Appl. Surf. Sci. 450, 312–317. 10.1016/j.apsusc.2018.04.139

[B16] Gao K. Chang Q. Wang B. Zhou N. Qing T. (2018b). The purification and tribological property of the synthetic magnesium silicate hydroxide modified by oleic acid. Lubr. Sci. 2018, 377–385. 10.1002/ls.1428

[B17] Grzybek T. Pietrzak R. Wachowska H. (2004). The comparison of oxygen and sulfur species formed by coal oxidation with O_2_/Na_2_CO_3_ or peroxyacetic acid solution. XPS Studies. Energy Fuels 18, 804–809. 10.1021/ef030153i

[B18] Guinneton F. Monnereau O. Argeme L. Stanoi D. Socol G. Mihailescu I. N.. (2005). PLD thin films obtained from CrO_3_ and Cr_8_O_2_1 targets. Appl. Surf. Sci. 247, 139–144. 10.1016/j.apsusc.2005.01.073

[B19] Hassan A. M. Al-Bsharat A. S. (1996). Influence of burnishing process on surface roughness, hardness, and microstructure of some non-ferrous metals. Wear 199, 1–8. 10.1016/0043-1648(95)06847-3

[B20] Hirano M. Shinjo K. (1993). Superlubricity and frictional anisotropy. Wear 168, 121–125. 10.1016/0043-1648(93)90207-3

[B21] Honma T. Sato R. Benino Y. Komatsu T. Dimitrov V. (2000). Electronic polarizability, optical basicity and XPS spectra of Sb_2_O_3_-B_2_O_3_ glasses. J. Non Cryst. Solids 272, 1–13. 10.1016/S0022-3093(00)00156-3

[B22] Hu J. J. Bultman J. E. Zabinski J. S. (2006). Microstructure and lubrication mechanism of multilayered MoS_2_/Sb_2_O_3_ thin films. Tribol. Lett. 21, 169–174. 10.1007/s11249-006-9035-6

[B23] Huang Y. Ruiz P. (2005). Antimony dispersion and phase evolution in the Sb_2_O_3_-Fe_2_O_3_ system. Phys. Chem. B 109, 22420–22425. 10.1021/JP053785+ 16853920

[B24] Jantschner O. Field S. K. Holec D. Fian A. Music D. Schneider J. M.. (2015). Origin of temperature-induced low friction of sputtered Si-containing amorphous carbon coatings. Acta Mater. 82, 437–446. 10.1016/j.actamat.2014.09.030

[B25] Jantschner O. Field S. K. Music D. Terziyska V. L. Schneider J. M. Munnik F.. (2014). Sputtered Si-containing low-friction carbon coatings for elevated temperatures. Tribol. Int. 77, 15–23. 10.1016/j.triboint.2014.04.006

[B26] Jha A. K. Prasad K. Prasad K. (2009). A green low-cost biosynthesis of Sb2O3 nanoparticles. Biochem. Eng. J. 43, 303–306. 10.1016/j.bej.2008.10.01619844916

[B27] Jia K. Li Y. Q. Fischer T. E. Gallois B. (1995). Tribology of diamond-like carbon sliding against itself, silicon nitride, and steel. J. Mater. Res. 10, 1403–1410. 10.1557/JMR.1995.1403

[B28] Kawai S. Benassi A. Gnecco E. Söde H. Pawlak R. Feng X.. (2016). Superlubricity of graphene nanoribbons on gold surfaces. Science 351, 957–961. 10.1126/science.aad3569 26917767

[B29] Kumar P. R. Prasad N. Veillon F. Prellier W. (2020). Raman spectroscopic and magnetic properties of europium doped nickel oxide nanoparticles prepared by microwave-assisted hydrothermal method. J. Alloys Compd. 2020:157639. 10.1016/j.jallcom.2020.157639

[B30] Li H. Wang J. Gao S. Chen Q. Peng L. Liu K.. (2017). Superlubricity between MoS_2_ monolayers. Adv. Mater. 29:1701474. 10.1002/adma.201701474 28497859

[B31] Li H. Wood R. J. Rutland M. W. Atkin R. (2014). An ionic liquid lubricant enables superlubricity to be switched on *in situ* using an electrical potential. Chem. Commun. 50, 4368–4370. 10.1039/c4cc00979g 24643511

[B32] Liu Z. Yang J. Grey F. Liu J. Z. Liu Y. Wang Y.. (2012). Observation of microscale superlubricity in graphite. Phys. Rev. Lett. 108:205503. 10.1103/PhysRevLett.108.205503 23003154

[B33] Martin J. M. Donnet C. Le Mogne T. Epicier T. (1993). Superlubricity of molybdenum disulphide. Phys. Rev. B 48, 10583–10586. 10.1103/PhysRevB.48.10583 10007345

[B34] Morent R. De Geyter N. Leys C. Gengembre L. Payen E. (2008). Comparison between XPS- And FTIR-analysis of plasma-treated polypropylene film surfaces. Surf. Interface Anal. 40, 597–600. 10.1002/sia.2619

[B35] Naidu B. S. Pandey M. Sudarsan V. Vatsa R. K. Tewari R. (2009). Photoluminescence and Raman spectroscopic investigations of morphology assisted effects in Sb_2_O_3_. Chem. Phys. Lett. 474, 180–184. 10.1016/j.cplett.2009.04.050

[B36] Nan J. Yang Y. Lin Z. (2006). *In situ* photoelectrochemistry and Raman spectroscopic characterization on the surface oxide film of nickel electrode in 30 wt.% KOH solution. Electrochim. Acta 51, 4873–4879. 10.1016/j.electacta.2006.01.031

[B37] Samanta S. Yadav R. Kumar A. Kumar Sinha A. Srivastava R. (2019). Surface modified C, O co-doped polymeric g-C_3_N_4_ as an efficient photocatalyst for visible light assisted CO_2_ reduction and H_2_O_2_ production. Appl. Catal. B Environ. 259:118054. 10.1016/j.apcatb.2019.118054

[B38] Scharf T. W. Kotula P. G. Prasad S.V. (2010). Friction and wear mechanisms in MoS_2_/Sb_2_O_3_/Au nanocomposite coatings. Acta Mater. 58, 4100–4109. 10.1016/j.actamat.2010.03.040

[B39] Shirani A. Joy T. Rogov A. Lin M. Yerokhin A. Mogonye J. E.. (2020). PEO-chameleon as a potential protective coating on cast aluminum alloys for high-temperature applications. Surf. Coat. Technol. 397:126016. 10.1016/j.surfcoat.2020.126016

[B40] Veres M. Koós M. Orsós N. Tóth S. Füle M. Mohai M.. (2006). Incorporation of Si in a-C:Si:H films monitored by infrared excited Raman scattering. Diam. Relat. Mater. 15, 932–935. 10.1016/j.diamond.2005.10.051

[B41] Wang B. Chang Q. Gao K. Wen X. Bai P. Tian Y. (2020). Superlow wear realizable tribofilms from lubricant oil containing hydrothermally synthesized magnesium silicate hydroxide/carbon core–shell nanoplates. Langmuir. 37, 240–248. 10.1021/acs.langmuir.0c02845 33356284

[B42] Wang W. Xie G. Luo J. (2018a). Black phosphorus as a new lubricant. Friction 6, 116–142. 10.1007/s40544-018-0204-z

[B43] Wang W. Xie G. Luo J. (2018b). Superlubricity of black phosphorus as lubricant additive. ACS Appl. Mater. Interfaces 10, 43203–43210. 10.1021/acsami.8b14730 30419751

[B44] Yu H. Xu Y. Shi P. Wang H. Wei M. Zhao K.. (2013). Microstructure, mechanical properties and tribological behavior of tribofilm generated from natural serpentine mineral powders as lubricant additive. Wear 297, 802–810. 10.1016/j.wear.2012.10.013

[B45] Yu H. L. Xu Y. Shi P. J. Wang H. M. Zhao Y. Xu B. S.. (2010). Tribological behaviors of surface-coated serpentine ultrafine powders as lubricant additive. Tribol. Int. 43, 667–675. 10.1016/j.triboint.2009.10.006

[B46] Zabinski J. S. Donley M. S. McDevitt N. T. (1993). Mechanistic study of the synergism between Sb_2_O_3_ and MoS_2_ lubricant systems using Raman spectroscopy. Wear 165, 103–108. 10.1016/0043-1648(93)90378-Y

[B47] Zabinski J. S. Donley M. S. Walck S. D. Schneider T. R. McDevitt N. T. (1995). The effects of dopants on the chemistry and tribology of sputter-deposited MoS_2_ films. Tribol Trans 38, 894–904. 10.1080/10402009508983486

[B48] Zeng D. W. Xie C. S. Zhu B. L. Song W. L. (2004). Characteristics of Sb_2_O_3_ nanoparticles synthesized from antimony by vapor condensation method. Mater. Lett. 58, 312–315. 10.1016/S0167-577X(03)00476-2

[B49] Zeng Q. Yu F. Dong G. (2013). Superlubricity behaviors of Si_3_N_4_/DLC Films under PAO oil with nano boron nitride additive lubrication. Surf. Interface Anal. 45, 1283–1290. 10.1002/sia.5269

[B50] Zhang D. Li S. Zuo X. Guo P. Ke P. Wang A. (2020). Structural and mechanism study on enhanced thermal stability of hydrogenated diamond-like carbon films doped with Si/O. Diam. Relat. Mater. 108:107923. 10.1016/j.diamond.2020.107923

[B51] Zhao F. Bai Z. Fu Y. Zhao D. Yan C. (2012). Tribological properties of serpentine, La(OH)_3_ and their composite particles as lubricant additives. Wear 288, 72–77. 10.1016/j.wear.2012.02.009

