# Supplementary material for: Macroscale Superlubricity Accomplished by Sb2O3-MSH/C Under High Temperature
Source: Front Chem. 2021 Apr 15;9:667878. doi: 10.3389/fchem.2021.667878 (PMC8083055; doi:10.3389/fchem.2021.667878)
Supplement: Supplementary file 1 [file Data_Sheet_1.docx]

Supplementary Information

Macroscale superlubricity accomplished by Sb_2_O_3_-MSH/C under high temperature

GAO Kai^1^, WANG Bin^2^, Asghar Shirani^3^, CHANG Qiuying^1^, Diana Berman^3, *^

*1．Beijing Jiaotong University, School of Mechanical, Electronic and Control Engineering, Beijing 100044, China*

*2. State Key Laboratory of Tribology, Tsinghua University, Beijing 100084, China*

*3. Materials Science and Engineering Department, University of North Texas, Denton, Texas 76203, United States*

*Corresponding author: Diana Berman at [diana.berman@unt.edu](mailto:diana.berman@unt.edu)

*
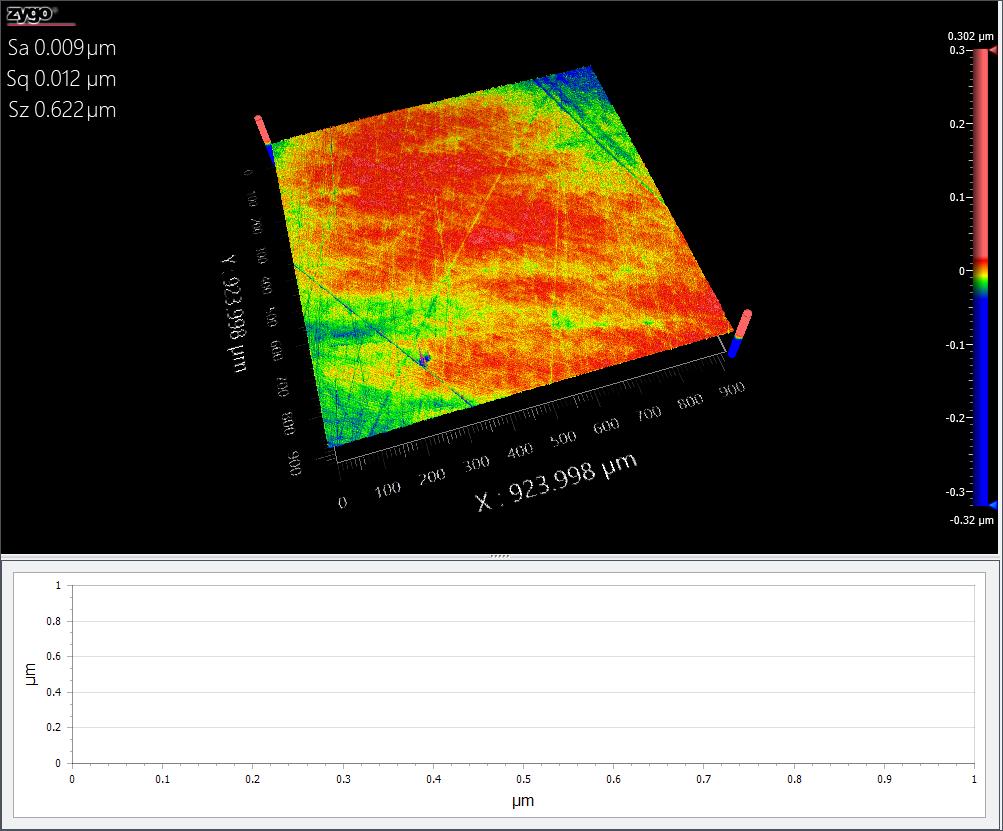
*

*Fig. S1 The roughness of the polished substrate before the burnishing process*

*
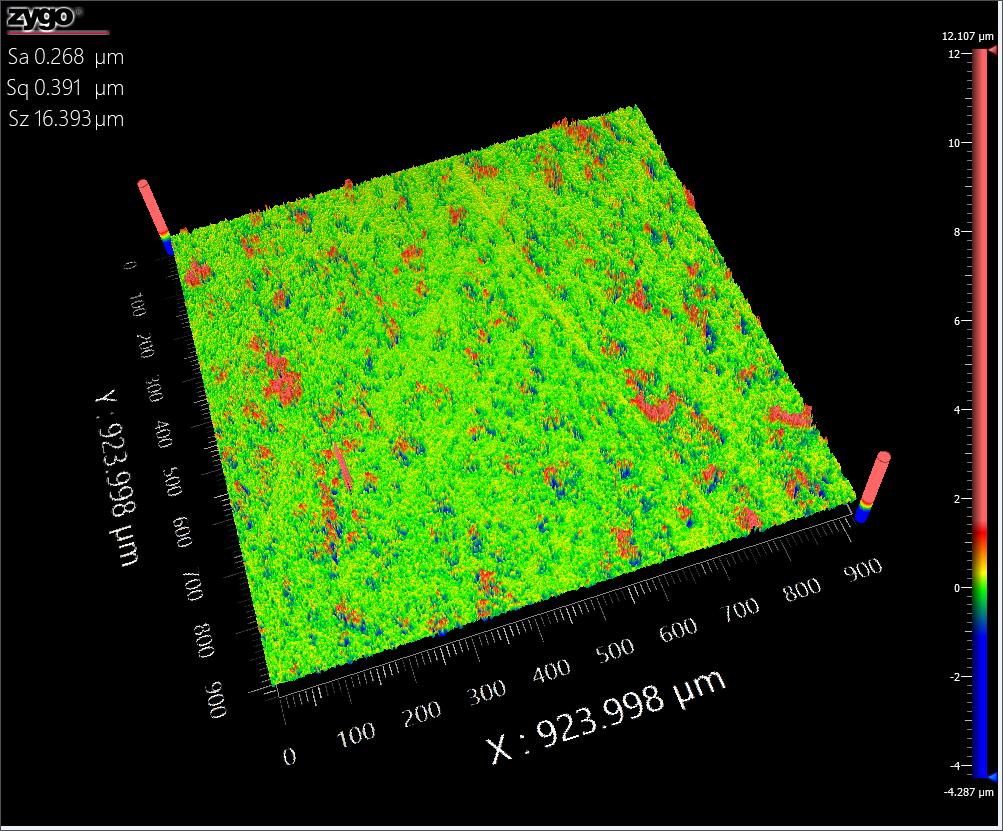
*

*Fig. S2 The roughness of the prepared sample after the burnishing process*


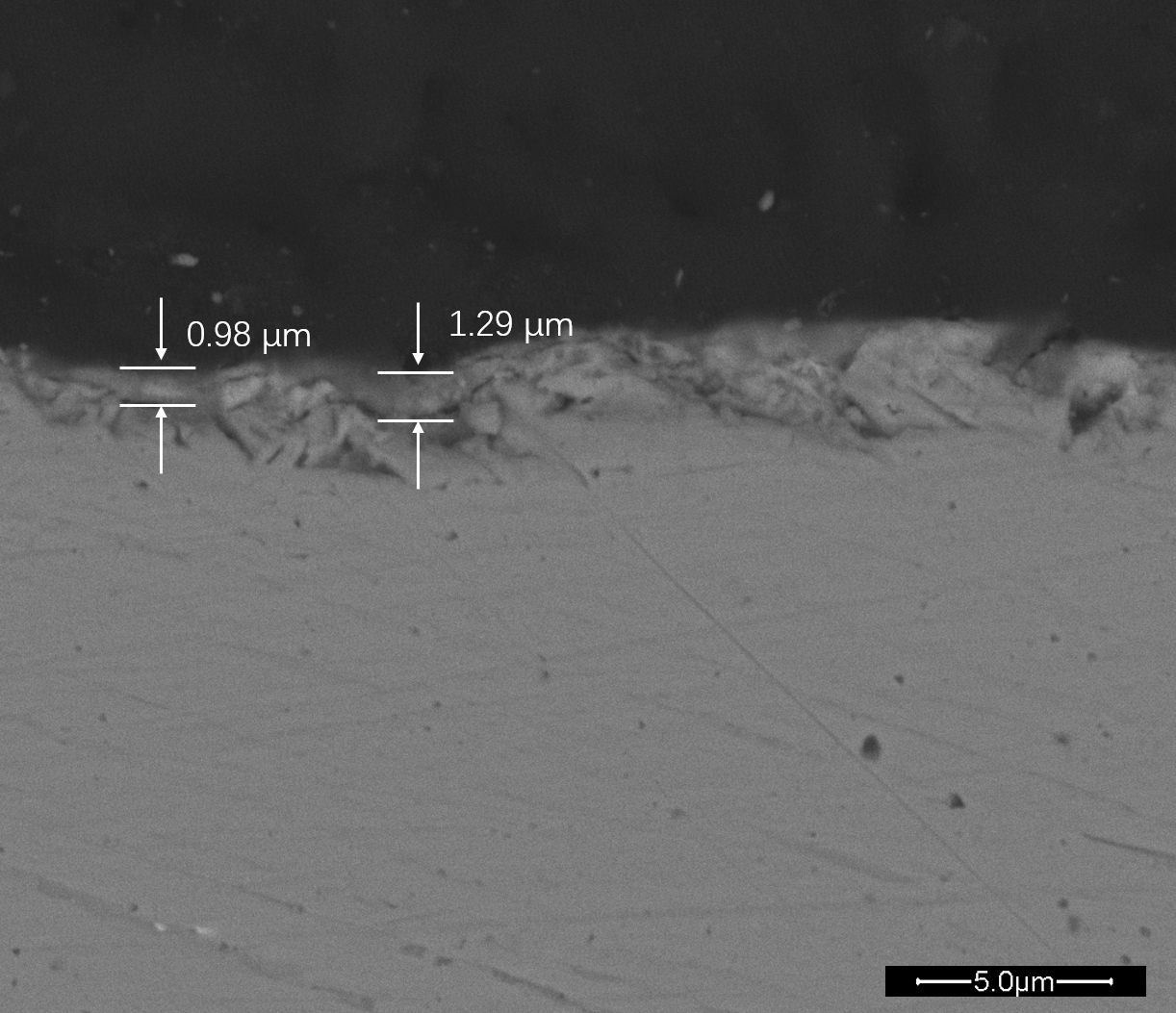


*Fig. S3 The thickness of the film after the burnishing process*


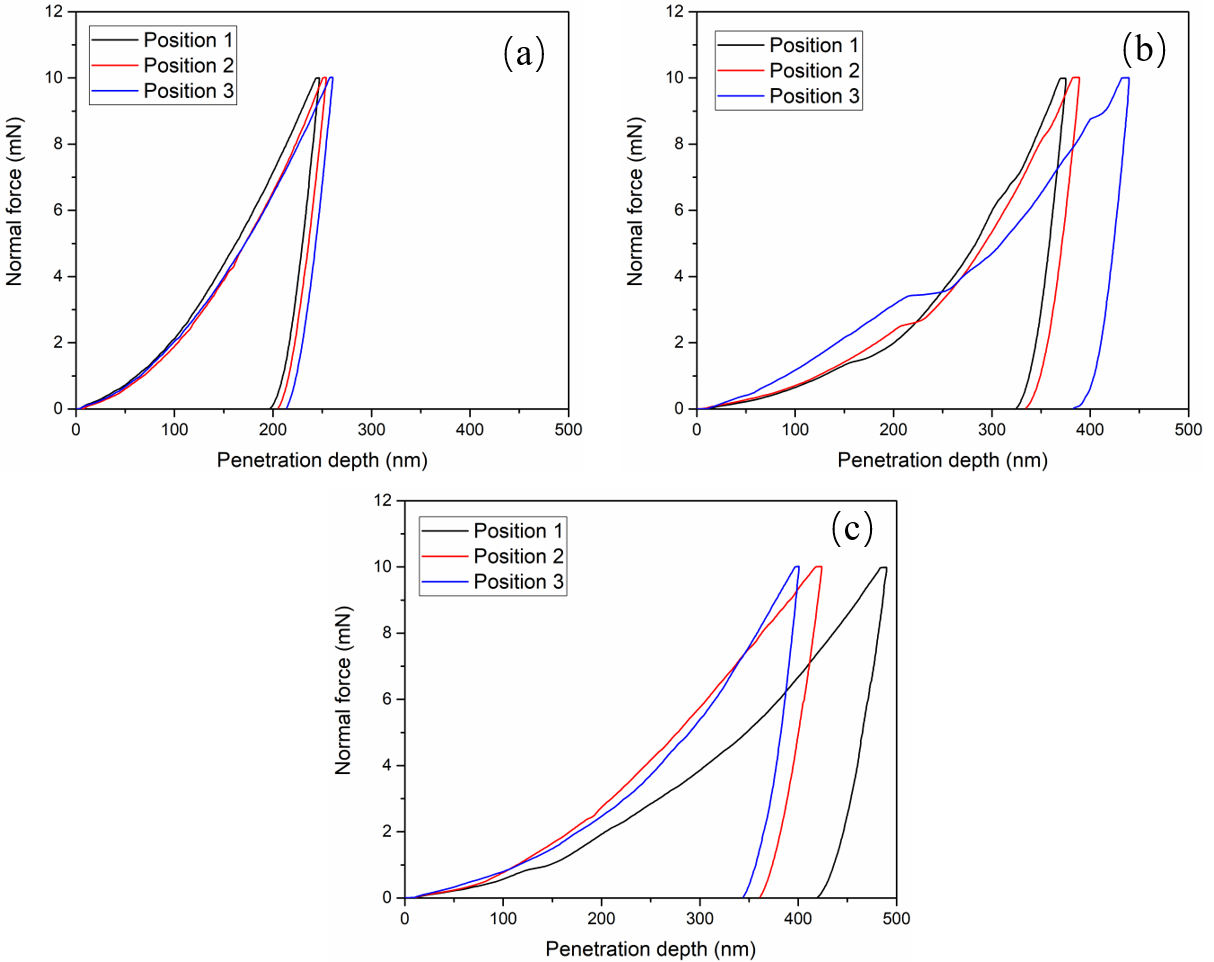


*Fig. S4 Load-penetration depth curves of (a)* *inconel substrate, (b) Sb2O3 and (c) Sb2O3-MSH/C film, tested by a* *nanoindentation tester (Berkovich B-T56)*


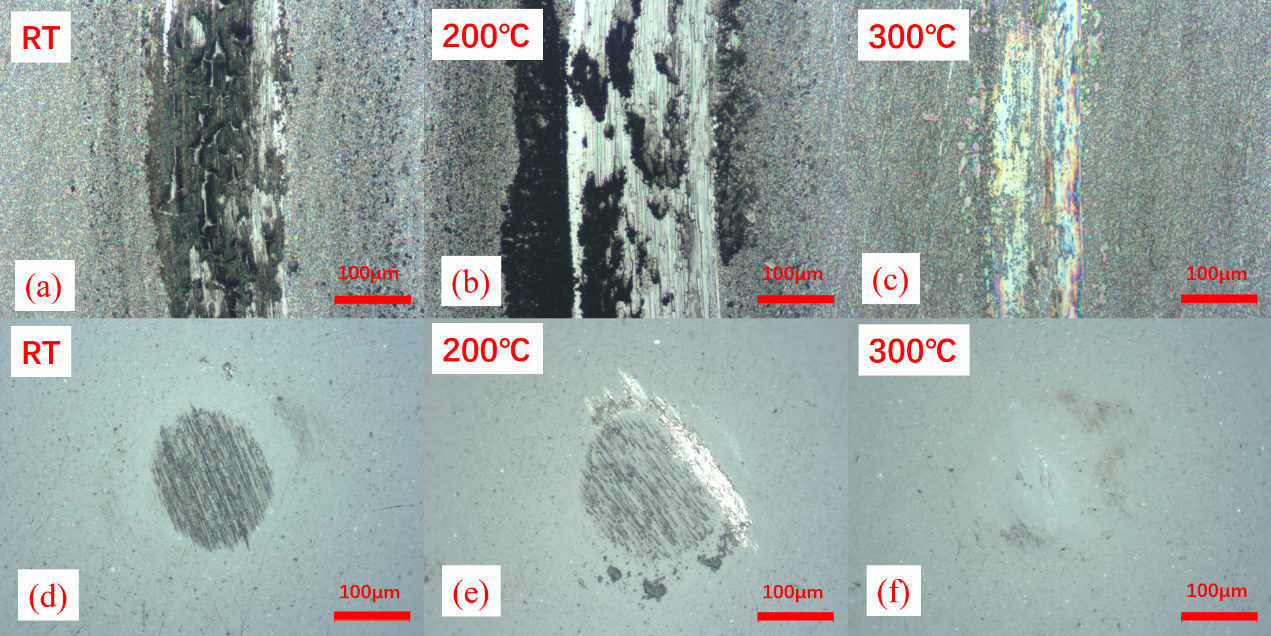


*Fig. S5 The optical images of (a)~(c) worn tracks, (d)~(f) corresponding ball worn surfaces for the Sb_2_O_3_ sample after the tribotests performed under different temperature conditions*


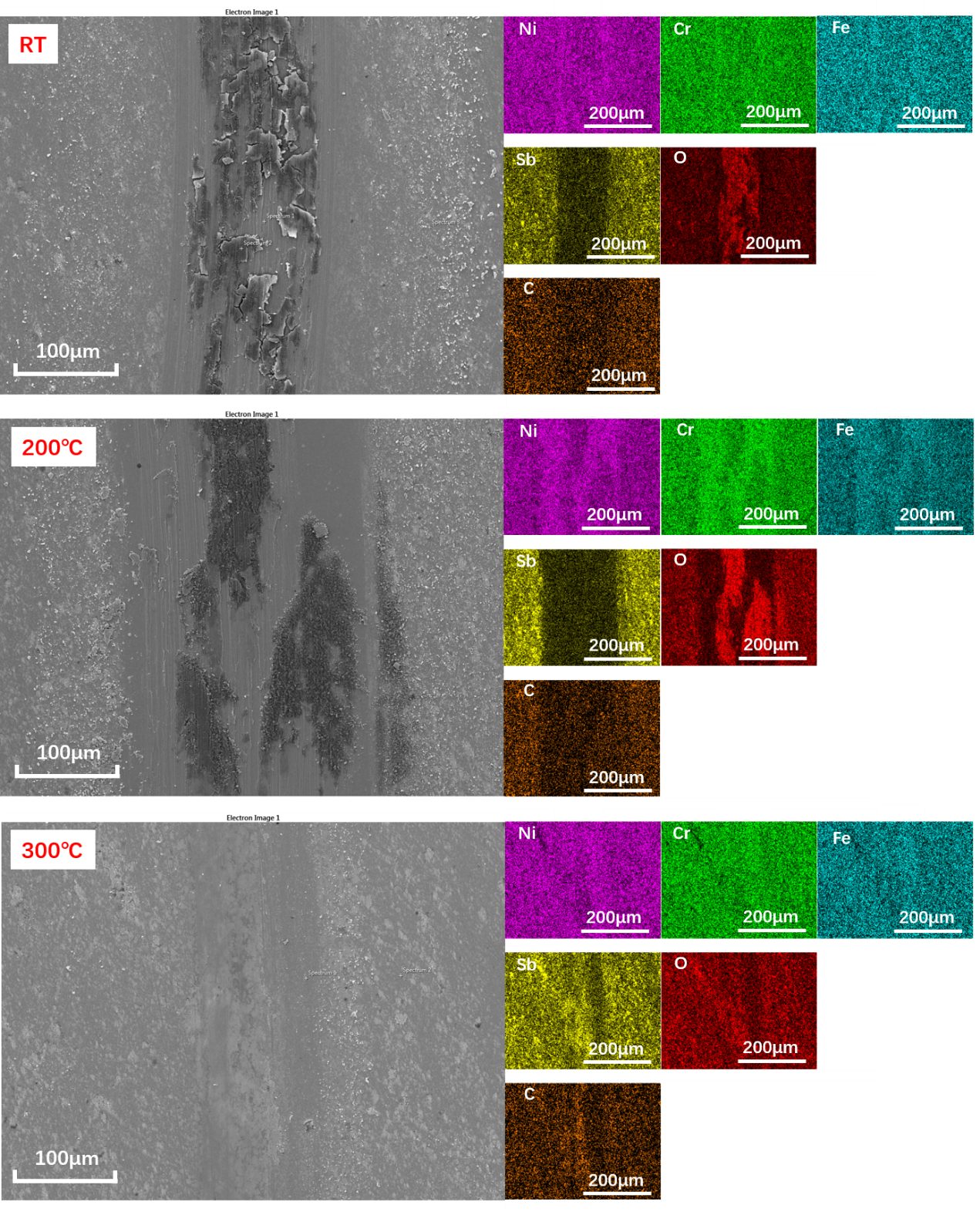


*Fig. S6 The SEM and the corresponding EDS elemental mapping images of Sb_2_O_3_ wear track areas after the tests performed at different temperatures*


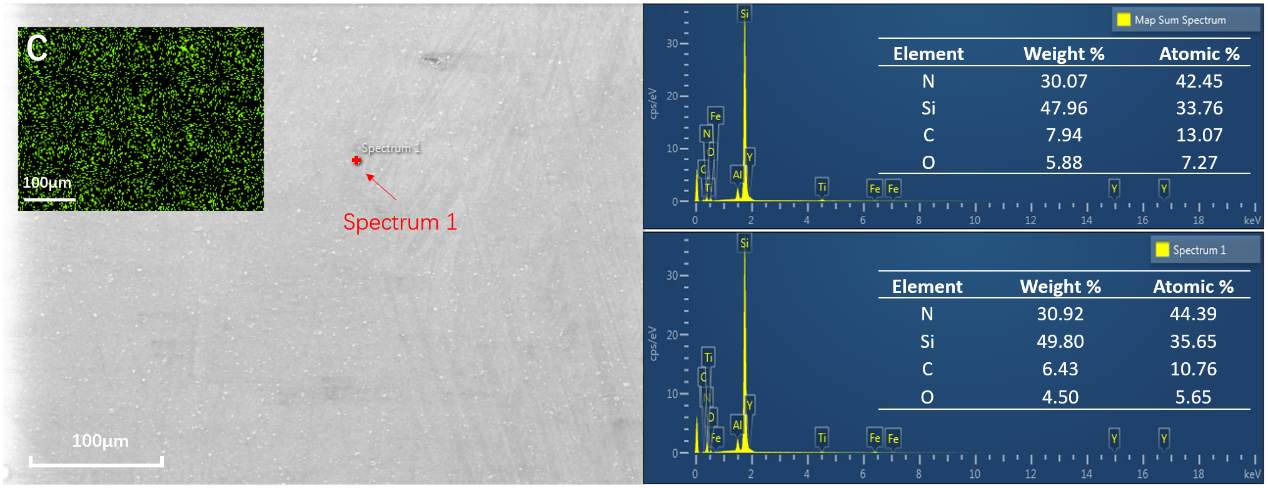


*Fig. S7 The EDS elemental contents of the wearmarks for the silicon nitride balls after the tests performed at 300 ºC for Sb_2_O_3_-MSH/C samples*
